# Copper coordination modulates prion conversion and infectivity in mammalian prion proteins

**DOI:** 10.1080/19336896.2022.2163835

**Published:** 2023-01-03

**Authors:** Giuseppe Legname

**Affiliations:** Laboratory of Prion Biology, Department of Neuroscience, Scuola Internazionale Superiore Di Studi Avanzati (SISSA), Trieste, Italy

**Keywords:** prion, prion protein, octarepeats, histidines, infectivity

## Abstract

In mammals the cellular form of the prion protein (PrP^C^) is a ubiquitous protein involved in many relevant functions in the central nervous system. In addition to its physiological functions PrP^C^ plays a central role in a group of invariably fatal neurodegenerative disorders collectively called prion diseases. In fact, the protein is a substrate in a process in which it converts into an infectious and pathological form denoted as prion. The protein has a unique primary structure where the unstructured N-terminal moiety possesses characteristic sequences wherein histidines are able to coordinate metal ions, in particular copper ions. These sequences are called octarepeats for their characteristic length. Moreover, a non-octarepeat fifth-copper binding site is present where copper coordination seems to control infectivity. In this review, I will argue that these sequences may play a significant role in modulating prion conversion and replication.

## Introduction

The cellular form of the prion protein (PrP^C^) is a physiological and ubiquitous molecule present in all mammal tissues [1]. It is particularly abundant in the central nervous system (CNS) where it is involved in the pathogenesis of various neurodegenerative diseases [2], known as transmissible spongiform encephalopathies (TSEs). The protein is either the substrate for the conversion, replication and accumulation into a pathogenic isoform denominated prion or PrP^Sc^ [3], or it may also serve as a promiscuous receptor for amyloidal forms of other proteins involved in neurodegenerative disorders [4]. Indeed, PrP^C^ has been found to interact and facilitate the uptake of various amyloid structures of proteins such as alpha-synuclein, tau and more recently the TAR DNA-binding protein 43 or TDP-43 [5–7]. The cellular prion protein, PrP^C^, is involved in different functions within the CNS, which include, among others, NMDAR regulation and neuritogenesis [8,9]. The protein has also been found involved in regulating the direction of synaptic plasticity in the hippocampus through protein kinase A [10]. For the many functions carried out by PrP^C^, the protein can be defined as a pleiotropic protein [1].

## Prion protein

The primary structure of PrP^C^ can be divided into an N-terminal unstructured, intrinsically disordered protein and well-structured and defined C-terminal moiety. At the amino terminal end an ER-localization sequence is found at residues 1 to 22 and the first amino acid, a lysine, starts at position 23. The mature sequence terminates at position 231 with a serine. The latter is further modified by the insertion of a glycosylphophatidylinositol group (by means of another processed signal sequence in position 232–254), which in turn anchors the protein to the outer leaflet of the plasma membrane [1].

In the mature form the protein is composed of either 208 or 209 amino acids (depending whether mouse or human sequences, respectively) with unique feature in its primary structure [11].

As mentioned above the N-terminal moiety is unstructured and it is characterized by the presence of four unique octapeptide repeat sequences (P**H**GGGWGQ) where the highlighted (in bold) histidine (His) within the sequence carries out a major function of coordinating divalent anions such as copper and to a lesser extent zinc [12,13]. These octapeptides structures are followed by a hydrophobic domain, with two main features: a so-called non-octarepeat fifth-copper binding site (95-**H**NQWNKPSKPKTNLK**H**-110) and a characteristic palindromic sequence (111-AGAAAAGA-119), which seems important for conversion of PrP^C^ to PrP^Sc^ [14]. The non-octarepeat fifth-copper binding site contains two highlighted (in bold) hystidine (His) residues which both coordinate mostly copper ions [15].

The structured part of PrP^C^ comprises three alpha-helices and three short beta-sheets. The first two beta-sheets (beta0 and beta1) precede the first alpha-helix, then a second beta-sheet (beta2) anticipates the last two helices [16]. The latter contain a single disulphide bridge and two sites for N-glycosylation [17].

**Figure 1.**
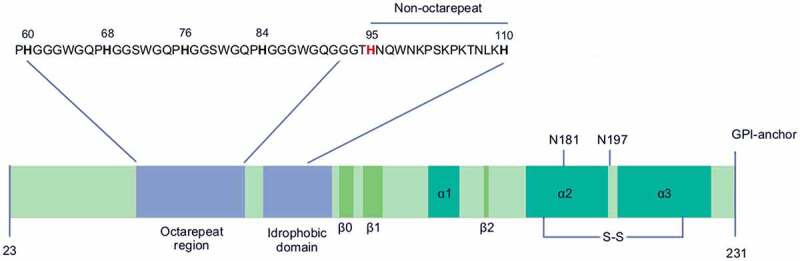
A cartoon representation of the primary structure of the mature mouse PrP^C^. The primary structure of the mature mouse PrP^C^ is shown. The highlighted amino acid sequences are shown of the N-terminal part including the octarepeats and the non-octarepeat fifth-copper binding site. The histidine residues involved in copper coordination are shown in bold either in red or in black. The single disulphide link and the two glycosylation sites are shown at the C-terminal domain.

All these PrP^C^ features are shown schematically in Figure 1.

## Copper coordination

Of the many unique characteristics of PrP^C^, certainly the distinctive capacity of coordinating copper ions has attracted much attention. Since the octapeptide repeat sequences and the non-octarepeat fifth-copper binding site are unique traits within the molecule consequently their role in both physiology and pathology has been explored in depth. Following the striking evidence that PrP^C^ alters copper content *in vivo* [18], the first report accounting for PrP^C^ binding to copper(II) ions was published about 25 years ago [19]. In this paper, using biophysical techniques such as near-UV circular dichroism (CD) and tryptophan (Trp) fluorescence spectroscopy, the authors offered evidence that copper(II) [or Cu(II)] induces conformational changes of PrP and indicate that additional equilibrium dialysis experiments are consistent with a stoichiometry of 2 ions per molecule. Only few years later by means of electron paramagnetic resonance (EPR) the ratio between copper(II) and octarepeat is found as 1:1 regardless the number of repeats present [20]. The atomic details of the coordination of copper within a single octarepeat sequence has been resolved indicating the direct involvement of glycine residues (Gly), besides the histidine, which revealed equatorial coordination by the histidine imidazole, two deprotonated glycine amides, a glycine carbonyl, along with an axial water bridging the tryptophan (Trp) indole [13]. Shortly after these seminal studies, another important step forward into the understanding of copper coordination in full-length PrP^C^ indicated the relevance of the non-octarepeat fifth-copper binding site in addition to the octapeptide repeat sequences [15].

**Figure 2.**
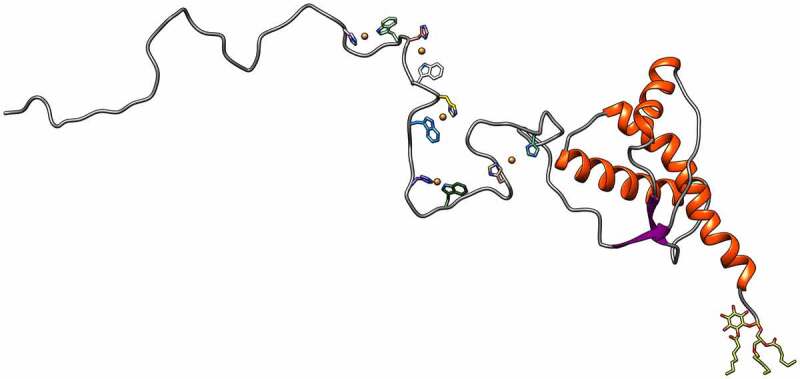
The three-dimensional (3D) structure of PrP^C^. The human 3D structure of PrP^C^ is shown in which the unstructured N-terminal domain is presented as a model of copper coordination. The C-terminal domain is represented based on structures solved by NMR.

The complete coordination structures of copper(II) binding to the various PrP sites have been reviewed recently [21]. The three-dimensional structure of PrP^C^ showing the coordination of copper ions at the unstructured N-terminal domain is shown in Figure 2.

## Physiology

Therefore, it would be difficult to consider PrP^C^ simply an apoprotein, without the coordination of copper. In other words, it seems that PrP^C^ requires the presence of copper in order to carry out its functions. The importance of copper coordination has been well-studied at least for its neuritogenesis function [22]. In addition, copper coordination in the whole prion protein is key in the regulation of NMDR receptor where the Cu(II) and Cu(I) coordination play a major role [8]. Instead, the role of copper in modulating prion conversion, replication and infectivity has been controversial for some time. Indeed, different reports indicate copper coordination to either promote or inhibit prion conversion [23,24]. In this review, I will try to present recent evidence that support the role of copper coordination in modulating prion conversion and infectivity.

## Copper coordination and prion conversion and infectivity

In biological terms, prions are infectious proteins that lead to neurodegeneration in mammals. In laboratory animals, upon inoculation in the CNS or other routes, prions will lead to a given set of neuropathological and biochemical changes that will define the infectious proteins. The inoculation can occur in a variety of different tissues but then the pathological changes and incubation times may vary depending on the site of inoculation. Regardless the site of inoculation, the outcome will be neurodegeneration of the CNS.

Prion infectivity is defined by several criteria. First, any given prion specimen will lead to characteristic incubation times that depend on the prion itself (prion strain), the homology of PrP^C^ between the prion inoculum and the animal used in the experiment, and to the genetic background of the recipient laboratory animal (usually an inbred or outbred wild-type mouse strain, or transgenic mouse strains). The combination of these parameters for any given prion strain will harbour invariably similar incubation times, neuropathological changes and biochemical features over time. In fact, models of prion disorders in laboratory animal are the most reliable experimental representation of the human disease counterparts.

So, what does it make an infectious prion? At the molecular level, the structure of the prion is essential for its infectivity. Indeed, in human prion diseases there is a variety of molecular evidence about the nature of prions. For long time the neuropathological occurrence of prion protein amyloid deposits in the CNS was believed to be the main characteristic of prions. Nevertheless, several human prion disorders present neurodegeneration without prion amyloid deposits, thus implying that prion amyloid formation is a non-obligatory feature of these disorders. This is the case for some forms of fatal familial insomnia [25]. A prion can exist in an infectious form even without forming detectable amyloidal structure (see below for prion structures). Early studies on the required molecular features for infectious prion showed that the palindromic sequence (111-AGAAAAGA-119) within the N-terminal hydrophobic domain is essential for prion formation and conversion. Deletion mutants lacking the sequence AGAAAAGA did not convert and replicate using persistently scrapie-infected mouse neuroblastoma cells as a model system for prion replication [26,27].

Proteolytic processing of PrP has been known to provide further evidence of the relevance and importance of copper-PrP interactions because the resulting fragments could provide a high affinity binding site for Cu(II) [28].

Supporting the role of the non-octarepeat fifth-copper binding site in prion conversion, transgenic mice were generated with an amino acid replacement at residue H95 with a glycine, which lead to shorter incubation times upon inoculation with infectious prions compared to wild-type animals [29].

In addition, structural evidence has reinforced the crucial role of the hydrophobic domain in the formation of infectious prions [30].

Studies with synthetic prions, which offer a relevant molecular model of native prions, served as an important indication about the role of copper coordination and prion conversion and infectivity in laboratory animals. Using a transgenic mouse model for prion replication denoted as Tg9949, it was possible to establish that the octapeptide repeat sequences were dispensable for prion formation, conversion, accumulation and infectivity [31]. This model of transgenic mice expresses a truncated form of the prion protein as a transgene, where the sequence is between residues 89 to 230 (start at amino acid 89, which is a glycine). In this sequence, as previously shown by Flechsig and colleagues, the octapeptide repeat sequences are missing but the protein still does convert to an infectious prion [31,32]. After a series of studies that strengthen this evidence, both the non-octarepeat fifth-copper binding site and the palindromic sequence AGAAAAGA, indicate a clear role for the hydrophobic domain in the production of prions. In addition, complete deletion of both octapeptide repeat sequences and hydrophobic domain leads to a toxic molecule denoted as PrP121-230 [33].

Therefore, it could be argued that the minimal structure for infectious prion should start at the hydrophobic domain and should cover the remaining of the molecule, at least up to amino acid 145 [34].

Of note, there a series of human mutant prion proteins in which the octapeptide repeat sequences are expanded (up to nine) and the number of extra octapeptide repeat sequences inversely correlates with disease onset [35].

One important evidence for the involvement of copper coordination in determining an infectious prion came from structural characterization of mutant pathogenic prion proteins.

Structural studies on human PrP with pathological Q212P, a mutation linked to Gerstmann-Sträussler-Scheinker (GSS) syndrome, is of relevance [36,37]. Using synchrotron radiation-based X-ray absorption fine structure technique, Cu(II) and Cu(I) coordination geometries in the mutant Q212P were compared to those of the wild-type protein. Analysing the extended X-ray absorption fine structure and the X-ray absorption near-edge structure, highlighted changes in copper coordination induced by the mutation Q212P in both oxidation states. While in the wild-type protein the copper-binding site has the same structure for both Cu(II) and Cu(I), in the mutant the coordination sites drastically change from the oxidized to the reduced form of the copper ion. Copper-binding sites in the mutant confirmed the loss of short- and long-range interactions [37].

More recently, another important contribution came from findings that highly conserved histidines in the C-terminal domain are crucial to hold together both the N-terminal and C-terminal moieties of PrP^C^ through Cu(II) coordination [38].

These results clearly indicate that point mutations in the C-terminal portion of PrP could affect copper coordination at the N-terminus and in particular copper binding at the non-octarepeat fifth-copper binding site.

In addition, one should also consider that the N-terminal domain of PrP^C^ is involved in the interaction with cellular partners in carrying out its function and thereferore copper coordination could compete with these protein binding sites and/or promote the formation of ternary complexes [39].

In order to model these effects, mutant proteins bearing either H96 or H111 mutations (human numbering) should lead to PrP molecules structural distinct in the non-octarepeat fifth-copper binding site [40]. In fact, the involvement of H96 and H111 in the non-octarepeat fifth-copper binding site shows that Cu(II) occupancy may play a crucial role in determining PrP^C^ conformation. Indeed, altered Cu(II) coordination due to a mutation that abrogates copper coordination, a tyrosine in place of the histidine (H96Y), may lead to spontaneous PrP^Sc^ formation in cultured neuronal cells and accumulation in the acid compartments. My group has proposed a model whereby HuPrP coordinating copper with His96 and His111 in the non-octarepeat fifth-copper binding site is more resistant to prion conversion compared to a PrP^C^ molecule coordinating Cu(II) with one histidine [40,41]. This work has been further supported by using a series of different PrP from several specie polymorphisms and mutants [42].

## Conclusions and further prospective

In this brief review, I highlighted the current literature that deals with copper coordination in the prion protein and the role of this metal in prion conversion and replication. It seems that copper may play a crucial role in determining whether a prion protein molecule becomes a prion.

Recently, a major step forward in the field of prion biology has been the resolution of the first structures of infectious prions [43–49]. It would be important to unveil the role of copper in generating such diverse structures for different prion strains.

It would also be quite important to carry out further studies on different mutants in place of the histidines 96 and 111 (human numbering) or 95 and 110 (mouse numbering) in the non-octarepeat fifth-copper binding site, in order to further establish their role in rendering PrP an infectious prion. The role of the non-octarepeat fifth-copper binding site seems to be crucial in establishing the structural determinant that leads to the formation of prions.
